# Frequency of Vital Signs Monitoring and its Association with Mortality among Adults with Severe Sepsis Admitted to a General Medical Ward in Uganda

**DOI:** 10.1371/journal.pone.0089879

**Published:** 2014-02-28

**Authors:** Stephen B. Asiimwe, Samson Okello, Christopher C. Moore

**Affiliations:** 1 Department of Internal Medicine, Mbarara Regional Referral Hospital, Mbarara, Uganda; 2 Department of Epidemiology and Biostatistics, University of California San Francisco, San Francisco, California, United States of America; 3 Department of Internal Medicine, Mbarara University of Science and Technology, Mbarara, Uganda; 4 Department of Medicine, Division of Infectious Diseases and International Health, University of Virginia, Charlottesville, Virginia, United States of America; D'or Institute of Research and Education, Brazil

## Abstract

**Introduction:**

Optimal vital signs monitoring of patients with severe sepsis in resource-limited settings may improve outcomes. The objective of this study was to determine the frequency of vital signs monitoring of patients with severe sepsis and its association with mortality in a regional referral hospital in Uganda.

**Methods:**

We reviewed medical records of patients admitted to Mbarara Regional Referral Hospital in Southwestern Uganda with severe sepsis defined by the presence of infection plus ≥2 of the systemic inflammatory response syndrome criteria, and ≥1 organ dysfunction (altered mental state, hypotension, jaundice, or thrombocytopenia). We recorded frequency of vital signs monitoring in addition to socio-demographic, clinical, and outcome data. We analyzed the data using logistic regression.

**Results:**

We identified 202 patients with severe sepsis. The median age was 35 years (IQR, 25–47) and 98 (48%) were female. HIV infection and anemia was present in 115 (57%) and 83 (41%) patients respectively. There were 67 (33%) in-hospital deaths. The median monitoring frequency per day was 1.1 (IQR 0.9–1.5) for blood pressure, 1.0 (IQR, 0.8–1.3) for temperature and pulse, and 0.5 (IQR, 0.3–1.0) for respiratory rate. The frequency of vital signs monitoring decreased during the course of hospitalization. Patients who died had a higher frequency of vital signs monitoring (p<0.05). The admission respiratory rate was associated with both frequency of monitoring (coefficient of linear regression 0.6, 95% CI 0.5–0.8, p<0.001) and mortality (AOR 2.5, 95% CI 1.3–5.3, p = 0.01). Other predictors of mortality included severity of illness, HIV infection, and anemia (p<0.05).

**Conclusions:**

More research is needed to determine the optimal frequency of vital signs monitoring for severely septic patients in resource-limited settings such as Uganda.

## Introduction

Severe sepsis is a serious illness that occurs when infection leads to organ failure and frequently causes death, especially if it is not rapidly recognized and treated [1]. Application of recent guidelines that emphasize early goal-directed resuscitation, prompt antibiotic administration, oxygen therapy, inotropic medications, and blood transfusion can reduce mortality in resource-rich settings [2], [3]. Modifications have been recommended for resource-limited settings, such as sub-Saharan Africa (SSA), which shoulder the largest burden of sepsis, but their applicability has been questioned [4]–[6]. Close monitoring of patients with severe sepsis may improve outcomes by identifying patients at risk of deterioration which can lead to appropriate interventions [1], [3], [7], [8].

The surviving sepsis campaign's international guidelines for management of sepsis do not make specific recommendations on the frequency of vital signs monitoring [1]. However, in resource-rich settings, management of severe sepsis usually takes place in emergency departments or intensive care units where continuous monitoring is possible [9]. The initial management should be goal-directed and iterative, hence frequent monitoring is necessary to meet pre-set targets [1], [3]. In contrast, in resource-limited settings, severely septic patients are typically managed on general wards [7], [10]–[12]. For patients with severe sepsis, the Integrated Management of Adolescent and Adult Illness (IMAI) guidelines for district hospitals in resource-limited settings created by the World Health Organization (WHO) recommend administration of antibiotics in the first hour along with monitoring blood pressure (BP), pulse, and respiratory rate every 30 minutes to hourly in the first 6 hours of admission, and hourly to every 2 hours thereafter for the next 24 hours [13], [14]. Additionally, temperature should be measured every 6 hours. After the first 24 hours, vital signs monitoring in the post-resuscitation period is recommended 1 to 3 times daily.

The current frequency of vital sign monitoring of patients with severe sepsis and the applicability of the IMAI guidelines in resource-limited settings such as government referral hospitals in SSA is unknown. Therefore, our objective was to determine the frequency of vital signs monitoring and its association with mortality for patients with severe sepsis managed on the general ward of a regional referral hospital in Uganda.

## Materials and Methods

This was an observational cohort study using medical records from the adult medical ward of Mbarara Regional Referral Hospital (MRRH) in Southwestern Uganda. At MRRH, patients with severe sepsis are initially resuscitated in an emergency room before admission to the ward. Initial diagnoses are made by medical residents and then reviewed by faculty physicians. The facilities available at MRRH have been previously described, but in addition, in September 2012, a new emergency room was opened where patients could stay overnight after admission [11]. This study was carried out in July 2013 and was approved by the Institutional Review Board of Mbarara University of Science and Technology. Since this was a retrospective study using data obtained from hospital charts, the institutional review board waived the need for written informed consent from the participants.

We reviewed admission records from July, October, and November of 2012, and January and February of 2013. We did not review records from holiday periods or times when clinical studies were being conducted on the ward. All patients admitted with a diagnosis of infection during the study period were identified from the hospital admission register. Their medical charts were then screened to identify patients with severe sepsis. Severe sepsis was defined as the presence of infection plus ≥2 systemic inflammatory response syndrome criteria (pulse ≥90 beats/minute; respiratory rate ≥20 cycles/minute; temperature ≥38°C or ≤36°C; and white blood cell concentration ≥12,000 cells/cc or <4,000 cells/cc or >10% band forms), and at least 1 organ dysfunction (altered mental state, systolic blood pressure [SBP] <90 or diastolic BP [DBP] <60 mmHg, jaundice, or platelet count <100,000 cells/cc).

### Measurements

The primary predictor was the frequency of vital signs monitoring including BP, temperature, pulse, and respiratory rate. We abstracted all vital signs recordings available for the duration of each admission together with their associated date and time. We obtained clinical data including diagnosis, antibiotics and intravenous fluids prescribed, along with laboratory data including complete blood counts and HIV serostatus. Anemia was defined as a hemoglobin of <12 g/dL for men and <11.5 g/dL for women. Additional information obtained included sex, age, occupation, district of residence, and whether the patient was admitted from the community or from another hospital or clinic. We determined the severity of illness by the total number of dysfunctional organs and a CRB-65 score [11], [15]. The CRB-65 score (range 0–4) was determined by the aggregate of points accrued for confusion, respiratory rate ≥20, systolic BP<90 mmHg or diastolic BP≤60 mmHg, and age ≥65. The primary outcome was in-hospital mortality. Other outcomes included self-discharge, referral to a higher level of care facility, readmission, and length of hospital stay.

### Analysis

The average frequency of vital signs monitoring was calculated as the total count for each recording of vital signs divided by days in hospital. The association of monitoring frequency with mortality was determined using logistic regression (Stata 12, StataCorp, College Station, Texas, USA). Statistical significance was set at p<0.05. In multivariable models we adjusted for severity of illness as measured by a CRB-65 score, age, sex, anemia, and HIV status. We also assessed the association of post admission changes in blood pressure with mortality. A sample size of 200 patients was required to detect a reduction in mortality of 13% per unit increase in monitoring frequency given an expected mortality rate among severely septic patients of 33.8%, an alpha of 0.05, a beta of 0.2, and a penalty of 0.3 for including other predictors in the multivariable model [11], [16].

## Results

### Patient characteristics at admission

**Figure 1:**
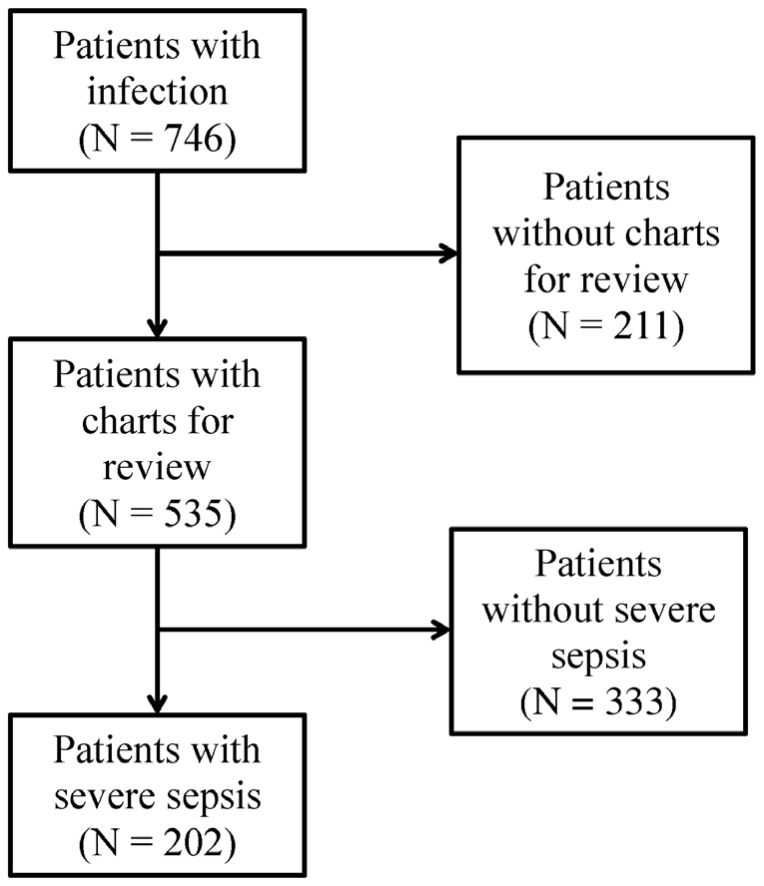
Flow diagram of study enrollment and analysis.

A total of 746 patients were admitted with an initial diagnosis of infection during the investigated time period. Of these, 211 (28%) charts were not available for review. From the 535 charts obtained, 202 (38%) patients had severe sepsis (Figure 1). The median age of the patients was 35 years (IQR, 25–47) and 98 (48%) were female. HIV infection was present in 115 (57%) patients and anemia was present in 83 (41%). The cardiovascular (123, 61%) and central nervous (53, 26%) systems were most frequently compromised. A broad-spectrum antibiotic was prescribed to 163 (81%) patients at admission (Table 1).

**Table 1:** Patient Characteristics at Admission.

Variable	
Sex, Female, N (%)	98 (48)
Referred from Other Centers, N (%)	67 (33)
Age, Median (IQR)	35 (25–47)
Occupation, N (%)	
Subsistence farmer	36 (29)
Self-employed	49 (39)
Professional	17 (14)
Others	24 (19)
Missing	76 (38)
Focus of Infection, N (%)	
Respiratory	97 (48)
Gastrointestinal	41 (20)
Central Nervous System	38 (19)
Unknown	14 (6.9)
Number of Organ Failures, N (%)	
1 Organ	167 (83)
2 Organs	33 (16)
≥3 Organs	2 (1.0)
Organs Failing, N (%)	
Cardiovascular	123 (61)
CNS	53 (26)
Hepatic	10 (5.0)
Hematologic	15 (7.4)
Anemia, N (%)	83 (41)
Wasted, N (%)	57 (28)
Broad Spectrum Antibiotic Prescribed, N (%)	163 (81)
HIV Infected, N (%)	115 (57)
White Cell Counts (×10^3^/cc), Median (IQR)	5.5 (2.8–10)
Hemoglobin (g/dL), Median (IQR)	9.2 (6.2–12)
Platelets (×10^3^/cc), Median (IQR)	117 (66–192)
Volume of fluids (L) prescribed at admission, Median (IQR)	2 (1–2)

### Outcomes

There were 67 (33%) in-hospital deaths, 108 (54%) discharges after improvement, 16 (8%) self-discharges, 7 (3.5%) referrals to a higher level of care facility, and 4 (2%) re-admissions within 30 days of discharge which led to 3 additional deaths. The median length of stay for all patients was 5 days (IQR, 3–9). There were 24 deaths (36%) in the first 24 hours, and 33 (49%) in the first 48 hours. The median length of hospitalization for those who died was 3 days (IQR, 1–7) compared to 6 days (IQR, 4–10) for those who survived to discharge (p<0.001). Independent predictors of mortality included severity of illness, HIV infection, anemia, a respiratory rate ≥20, and the presence of CNS dysfunction (Table 2).

**Table 2:** Predictors of mortality.

Variable	OR	95% CI	P	AOR	95% CI	P
Age	1.0	1.0–1.0	0.335	1.0	1.0–1.1	0.179
Sex male	1.1	0.6–2.1	0.653	1.8	0.9–3.5	0.120
Admission status						
Respiratory rate ≥20/min	4.5	1.5–14	0.008	4.5	1.4–15	0.012
MAP≤65 mmHg	1.3	0.7–2.4	0.345	1.0	0.5–2.1	0.929
Temperature ≤36°C	2.7	1.2–6.4	0.019	4.4	1.6–13	0.005
Heart Rate ≥100/min	1.2	0.6–2.3	0.705	1.3	0.6–2.7	0.544
Severity of illness#						
CRB-65 score≥2	2.9	1.0–8.8	0.055	2.2	1.1–4.5	0.026
≥1 organ failure	2.2	1.1–4.6	0.036	1.5	0.6–3.6	0.358
Type of organ failure						
CVS	Ref	-	-	-	-	-
CNS	2.2	1.1–4.2	0.024	2.7	1.2–6.0	0.017
Thrombocytopenia/jaundice	1.4	0.6–3.4	0.477	1.5	0.6–4.1	0.396
Monitoring frequency						
BP per day*	2.0	1.2–3.2	0.004	2.5	1.4–4.5	0.001
Temperature per day	1.5	0.9–2.5	0.122	2.0	1.0–3.7	0.038
Pulse per day	1.7	1.0–2.9	0.051	2.3	1.2–4.5	0.013
Respiratory rate per day	2.8	1.5–5.3	0.001	2.5	1.3–5.3	0.010
Change in MAP after admission						
MAP decrease ≥10 mmHg	2.5	1.0–6.3	0.048	-	-	-
MAP did not change	1.2	0.6–2.5	0.658	-	-	-
MAP increased ≥10 mmHg	Ref	-	-	-	-	-
Anemia	1.5	0.8–2.7	0.189	2.0	1.0–4.1	0.049
HIV infection	2.4	1.2–4.9	0.018	3.9	1.6–9.0	0.002

*Variables representing frequency of monitoring were included in multivariable logistic models sequentially and adjusted for severity of illness, age, sex, HIV status, and anemia.

# Colinear variables were not included in the same models.

### Frequency of vital signs monitoring and association with mortality

In the first 24 hours of admission, 193 (96%) patients had at least 1 BP measurement, and 153 (76%) had 2 BP measurements. However, only 29 (14%) had 3 BP measurements within the first 24 hours of admission. The median monitoring frequency per day of hospital stay was 1.1 (IQR 0.9–1.5) for BP, 1.0 (IQR, 0.8–1.3) for temperature and pulse, and 0.5 (IQR, 0.3–1.0) for respiratory rate. In both the unadjusted and multivariable-adjusted analyses, an increase in monitoring frequency was associated with higher odds of mortality regardless of the initial severity of illness (Table 2). In the unadjusted analysis, a unit increase in BP monitoring frequency was associated with a 2-fold increase in the odds of mortality (OR 2.0, 95% CI 1.2–3.2, p = 0.004). This association persisted after adjusting for HIV status, anemia, and severity of illness (AOR 2.5, 95% CI 1.4–4.5, p = 0.001).

**Figure 2:**
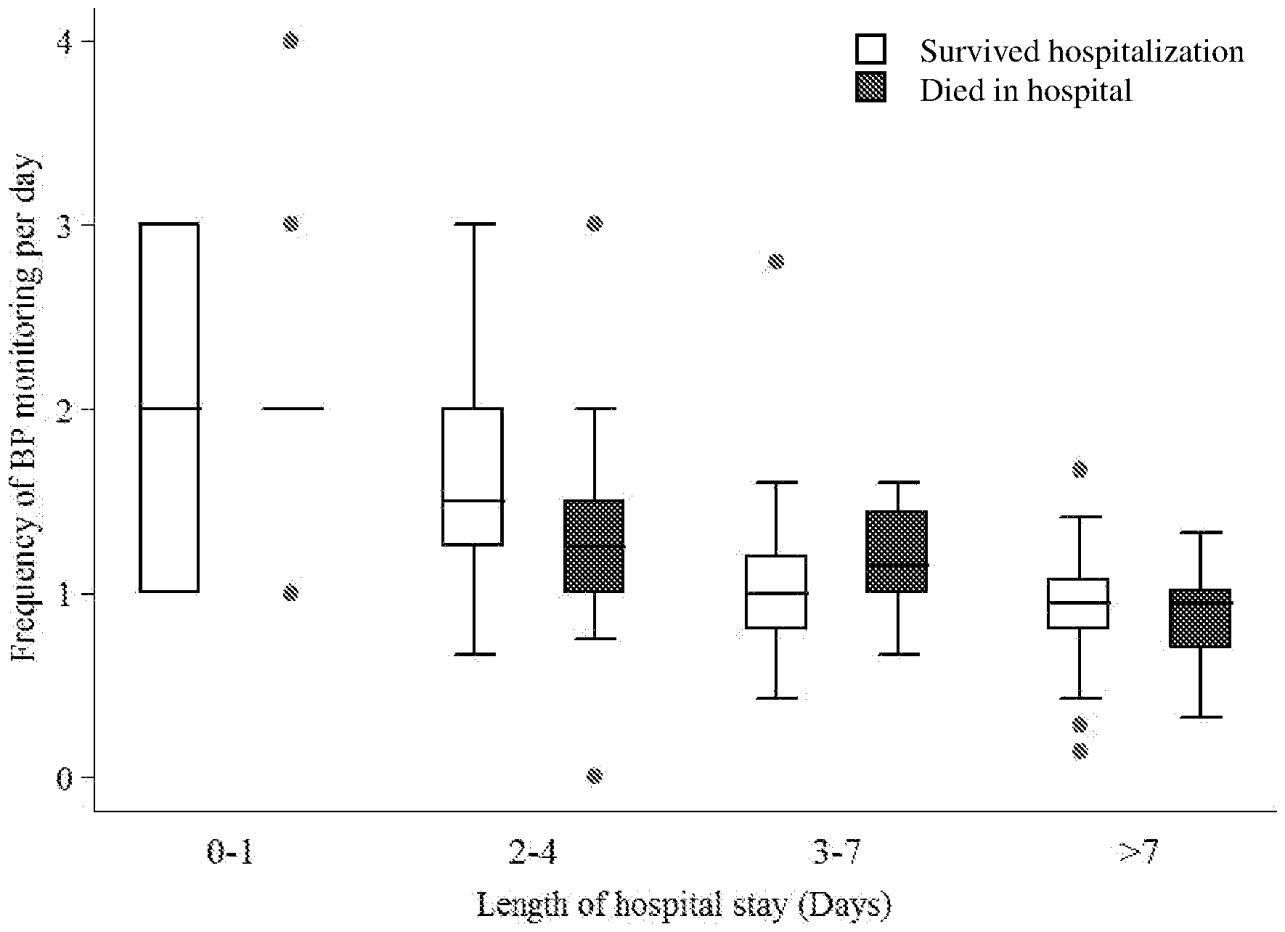
Frequency of blood pressure monitoring over the course of hospitalization according to final in-hospital vital status.

The frequency of BP monitoring was slightly higher (median 1.3 [IQR, 1.0–2.0]) for patients with >1 dysfunctional organs than for patients with 1 dysfunctional organ (median 1.1 [IQR, 0.9–1.5], p = 0.06). However, the median monitoring frequency for temperature, pulse, and respiratory rate did not vary according to the number of dysfunctional organs, and the frequency of monitoring for all vital signs did not vary according to CRB-65 score, focus of infection, or site of organ dysfunction. The admission respiratory rate did predict the frequency of vital signs monitoring with each unit increase in respiratory rate associated with a 0.6 unit increase in BP monitoring per day (coefficient of linear regression 0.6, 95% CI 0.5–0.8, p<0.001). The frequency of vital signs monitoring decreased throughout the duration of hospitalization (Figure 2).

### Post admission change in blood pressure and mortality

Repeat BP measurements on the day of admission were available for 147 patients. There was a decrease in MAP≥10 mmHg in 13 (15%) patients, a change in MAP<10 mmHg in either direction in 93 (63%) patients, and an increase in MAP≥10 mmHg in 35 (24%) patients. The admission MAP was higher (median 109 mmHg [90–120 mmHg]) for patients who had a subsequent decrease in MAP compared to patients who had a subsequent increase in MAP (Median 74 mmHg [50–85 mmHg], p = <0.001). Patients with a decrease in MAP after admission also received less fluid (median 0.75L, IQR 0–2) compared to patients with an increase in MAP (median 2L [IQR 2–3], p = 0.002).

Independent predictors of volume of fluid prescribed included severity of illness, anemia, focus of infection, and admission blood pressure. Patients with a CRB-65 score≥3 received an average of 1L more of fluid compared to patients with a CRB-65 score of 1 or 2 (coefficient of linear regression 1.1, 95% CI 0.3–1.9, p = 0.008). Patients with anemia received 0.6L less fluids than patients without anemia (coefficient of linear regression −0.6, 95% CI −1.0 to −0.2, p = 0.008). Patients with a gastrointestinal focus of infection also received about 0.6L more fluid than those with other foci (coefficient 0.6, 95% CI 0.1–1.2, p = 0.034).

## Discussion

Interventions are urgently needed to improve the recognition and treatment of severe sepsis in SSA which suffers the highest proportion of global sepsis and associated mortality [4], [6]. In the first study of its kind in SSA, we show that there was a paucity of vital signs monitoring which decreased over the course of hospitalization for adult patients admitted with severe sepsis to a regional referral hospital in Uganda. In resource-rich settings, early identification of deteriorating patients has been shown to improve outcomes through initiation of targeted interventions [1], [3], [17]. However, early identification requires frequent monitoring and the response to intravenous fluids and antibiotics requires follow-up evaluation [1], [13], [14]. In our study population, there was a high mortality rate suggesting that there were opportunities for additional interventions after admission which may have improved outcomes. The reasons for the infrequent monitoring and poor outcomes are complex and likely related to a low clinical staff-to-patient ratio as well as a lack of diagnostic and therapeutic resources.

A comprehensive response to clinical deterioration after admission for severe sepsis requires an afferent limb to identify the patient in possible crisis and to initiate an alert, and an efferent limb to rapidly evaluate and treat the patient. In resource-rich settings, this is often accomplished through automated track-and-trigger protocols that prompt notification of a rapid response team when a predetermined threshold of clinical risk is met [17]. However, even when such systems are in place, there is often a failure of the afferent limb of resuscitation [18]. Although we are not aware of any formalized rapid response teams in government referral hospitals in SSA, a previous study in Uganda showed that targeted monitoring and resuscitation of adults with severe sepsis at admission can improve outcomes [7]. We found that patients with lower admission BP received a greater volume of fluid resuscitation compared to those who had a higher admission BP. This finding suggests the existence of afferent and efferent resuscitation limbs which could be optimized to improve outcomes within the context of a resource-limited environment.

The association between increased frequency of vital signs monitoring and mortality in our study implies that there was clinical concern about patients who ultimately succumbed to severe sepsis. The severity of illness at admission, as measured by aggregate organ dysfunction or CRB-65 score, did not predict increased monitoring, but an increased respiratory rate at admission did predict both increased monitoring and mortality. Therefore, clinical concern may have originated from the admission respiratory rate. It is also possible that there was a causal relationship between increased vital signs monitoring and increased in-hospital mortality mediated through unnecessary or potentially harmful interventions [19], [20]. For example, the recently published FEAST trial revealed increased mortality from fluid resuscitation in children with severe sepsis in East Africa [21]. It is therefore possible that unmeasured interventions led to greater mortality among our study patients who were more frequently monitored. However, since the overall frequency of monitoring in our study was low, we believe it was sub-optimal rather than deleterious.

Increased vital signs monitoring should ideally strengthen the afferent limb of resuscitation and improve patient outcomes. In our study, the afferent limb of resuscitation was weakened by a median frequency of vital signs measurement of only ≤1.1 per day. In contrast, if the WHO's IMAI guidelines were followed, some patients would have had 24 BP measurements in the first day of admission [13], [14]. Given our findings, such a high frequency of vital signs monitoring may not be feasible in similarly resource constrained settings. In our study, only 14% of patients received 3 BP measurements in the first 24 hours.

Despite the relationship between monitoring frequency and mortality, there was a disconnect between the afferent and efferent limbs of resuscitation. For example, patients with a high respiratory rate had an increased risk of death and were monitored more frequently, but received no more fluids than the rest of the study population. Additionally, patients with lower admission BP or a high CRB-65 score received more fluid than the rest of the study population, but the volume of fluid given was small and they experienced similar mortality as those with higher admission BP. We interpret these findings to mean that clinicians were able to identify patients at risk of death but they were unable to prevent the progression to death through available interventions.

There are several possible reasons for the low frequency of vital signs monitoring in our study. First, intensive monitoring requires both human and material resources that are rarely available in resource-limited settings. Since we retrospectively diagnosed study patients with severe sepsis, clinicians may also not have been aware at the time of admission that patients had severe sepsis and required close monitoring. Even if patients were diagnosed with sepsis, those who presented with higher BPs were less likely to receive intravenous fluids. Clinicians may have thought these patients were clinically stable and did not require close monitoring or fluid resuscitation. Patients with normal BPs may have had other indicators of poor perfusion which were not detected in this resource-limited setting due in part to the lack of laboratory infrastructure [22]. For example, serum lactate is associated with mortality in severe sepsis independent of organ failure and overt shock [23]. A previous study from Uganda showed that point-of-care whole blood lactate testing predicted mortality in patients with severe sepsis [24].

Important predictors of mortality included CRB-65 score, number of organ failures, admission respiratory rate, anemia, and HIV infection.

This study did have limitations. Several patient charts were not available for review, so some patients admitted with severe sepsis during the investigated time period could have been missed. However, this was not expected to result in bias as the reason for missing charts was unlikely to be related to the frequency of vital signs monitoring or mortality. The low frequency of monitoring limited our ability to interpret the observed association between vital signs monitoring and mortality. Nonetheless, our findings reveal important deficiencies in the monitoring and resuscitation of patients with severe sepsis.

## Conclusions

Clinicians in areas with a high burden of infection and limited resources are frequently faced with critically ill patients with severe sepsis and septic shock. A high degree of suspicion for severe sepsis is needed for timely resuscitation of these patients. In this study, patients who eventually succumbed to sepsis raised a higher level of clinical concern at admission as evidenced by higher monitoring rates, but deficiencies in both the afferent and efferent limbs of resuscitation may have led to poor outcomes. A monitoring strategy that employs resource appropriate clinical early warning scores such as CRB-65 may help clinicians in austere environments to identify patients with severe sepsis that require rapid interventions. Accordingly, we recommend further study of predictors of severe sepsis at admission, the association between close monitoring and death, and the optimal monitoring rate for patients with severe sepsis. Although monitoring throughout hospitalization is important, given the precipitous rate of mortality observed in our study, a focus on the immediate post-admission period should be emphasized.

## References

[1] Dellinger RP, Levy MM, Rhodes A, Annane D, Gerlach H, et al (2013) Surviving sepsis campaign: international guidelines for management of severe sepsis and septic shock: 2012. Crit Care Med 41: 580–637. 23353941 10.1097/CCM.0b013e31827e83af

[2] Bouferrache K, Amiel JB, Chimot L, Caille V, Charron C, et al (2012) Initial resuscitation guided by the Surviving Sepsis Campaign recommendations and early echocardiographic assessment of hemodynamics in intensive care unit septic patients: a pilot study. Crit Care Med 40: 2821–2827. 22878678 10.1097/CCM.0b013e31825bc565

[3] Rivers E, Nguyen B, Havstad S, Ressler J, Muzzin A, et al (2001) Early goal-directed therapy in the treatment of severe sepsis and septic shock. N Engl J Med 345: 1368–1377. 11794169 10.1056/NEJMoa010307

[4] Adhikari NK, Fowler RA, Bhagwanjee S, Rubenfeld GD (2010) Critical care and the global burden of critical illness in adults. Lancet 376: 1339–1346. 20934212 10.1016/S0140-6736(10)60446-1 PMC7136988

[5] Jacob ST, West TE, Banura P (2011) Fitting a square peg into a round hole: are the current Surviving Sepsis Campaign guidelines feasible for Africa? Crit Care 15: 117. 21345262 10.1186/cc9981 PMC3222070

[6] Lozano R, Naghavi M, Foreman K, Lim S, Shibuya K, et al (2012) Global and regional mortality from 235 causes of death for 20 age groups in 1990 and 2010: a systematic analysis for the Global Burden of Disease Study 2010. Lancet 380: 2095–2128. 23245604 10.1016/S0140-6736(12)61728-0 PMC10790329

[7] Jacob ST, Banura P, Baeten JM, Moore CC, Meya D, et al (2012) The impact of early monitored management on survival in hospitalized adult Ugandan patients with severe sepsis: a prospective intervention study*. Crit Care Med 40: 2050–2058. 22564958 10.1097/CCM.0b013e31824e65d7 PMC3378757

[8] Oglesby KJ, Durham L, Welch J, Subbe CP (2011) ‘Score to Door Time’, a benchmarking tool for rapid response systems: a pilot multi-centre service evaluation. Crit Care 15: R180. 21794137 10.1186/cc10329 PMC3387623

[9] Schulman CS, Staul L (2010) Standards for frequency of measurement and documentation of vital signs and physical assessments. Crit Care Nurse 30: 74–76. 10.4037/ccn2010406 20515885

[10] Jacob ST, Moore CC, Banura P, Pinkerton R, Meya D, et al (2009) Severe sepsis in two Ugandan hospitals: a prospective observational study of management and outcomes in a predominantly HIV-1 infected population. PLoS One 4: e7782. 19907656 10.1371/journal.pone.0007782 PMC2771355

[11] Ssekitoleko R, Pinkerton R, Muhindo R, Bhagani S, Moore CC (2011) Aggregate evaluable organ dysfunction predicts in-hospital mortality from sepsis in Uganda. Am J Trop Med Hyg 85: 697–702. 21976575 10.4269/ajtmh.2011.10-0692 PMC3183780

[12] Ssekitoleko R, Jacob ST, Banura P, Pinkerton R, Meya DB, et al (2011) Hypoglycemia at admission is associated with inhospital mortality in Ugandan patients with severe sepsis. Crit Care Med 39: 2271–2276. 21666451 10.1097/CCM.0b013e3182227bd2 PMC3730257

[13] Jacob ST, Lim M, Banura P, Bhagwanjee S, Bion J, et al (2013) Integrating sepsis management recommendations into clinical care guidelines for district hospitals in resource-limited settings: the necessity to augment new guidelines with future research. BMC Med 11: 107. 23597160 10.1186/1741-7015-11-107 PMC3635910

[14] Jacob ST, Lim M, Banura P, Bhagwanjee S, Bion J, et al (2013) IMAI District Clinician Manual: Hospital Care for Adolescents and Adults. Guidelines for the Management of Illnesses with Limited Resources. World Health Organization Available: http://www.who.int/influenza/patient_care/IMAI_DCM/en/index.html.

[15] Bauer TT, Ewig S, Marre R, Suttorp N, Welte T (2006) CRB-65 predicts death from community-acquired pneumonia. J Intern Med 260: 93–101. 16789984 10.1111/j.1365-2796.2006.01657.x

[16] Demidenko E (2007) Sample size determination for logistic regression revisited. Stat Med 26: 3385–3397. 17149799 10.1002/sim.2771

[17] Winters BD, Weaver SJ, Pfoh ER, Yang T, Pham JC, et al (2013) Rapid-response systems as a patient safety strategy: a systematic review. Ann Intern Med 158: 417–425. 23460099 10.7326/0003-4819-158-5-201303051-00009 PMC4695999

[18] Trinkle RM, Flabouris A (2011) Documenting Rapid Response System afferent limb failure and associated patient outcomes. Resuscitation 82: 810–814. 21497982 10.1016/j.resuscitation.2011.03.019

[19] Kavanagh BP, Meyer LJ (2005) Normalizing physiological variables in acute illness: five reasons for caution. Intensive Care Med 31: 1161–1167. 16044251 10.1007/s00134-005-2729-7

[20] Webb SA, Young PJ, Bellomo R (2012) The “sweet spot” for physiological targets in critically ill patients. Crit Care Resusc 14: 253–255. 23230873

[21] Maitland K, Kiguli S, Opoka RO, Engoru C, Olupot-Olupot P, et al (2011) Mortality after fluid bolus in African children with severe infection. N Engl J Med 364: 2483–2495. 21615299 10.1056/NEJMoa1101549

[22] Petti CA, Polage CR, Quinn TC, Ronald AR, Sande MA (2006) Laboratory medicine in Africa: a barrier to effective health care. Clin Infect Dis 42: 377–382. 16392084 10.1086/499363

[23] Mikkelsen ME, Miltiades AN, Gaieski DF, Goyal M, Fuchs BD, et al (2009) Serum lactate is associated with mortality in severe sepsis independent of organ failure and shock. Crit Care Med 37: 1670–1677. 19325467 10.1097/CCM.0b013e31819fcf68

[24] Moore CC, Jacob ST, Pinkerton R, Meya DB, Mayanja-Kizza H, et al (2008) Point-of-care lactate testing predicts mortality of severe sepsis in a predominantly HIV type 1-infected patient population in Uganda. Clin Infect Dis 46: 215–222. 18171253 10.1086/524665

